# Les barrières à une bonne prise en charge des diabétiques dans les structures de première ligne de la province de Khouribga (MAROC)

**Published:** 2012-10-30

**Authors:** Samira Hassoune, Soufiane Badri, Samira Nani, Leila Belhadi, Abderrahmane Maaroufi

**Affiliations:** 1 Laboratoire d'Epidémiologie, Faculté de Médecine et de Pharmacie de Casablanca, Maroc; 2 Centre hospitalier universitaire Ibn Rochd de Casablanca, Maroc; 3 Service d'Endocrinologie, Centre hospitalier Baouafi, Casablanca, Maroc

**Keywords:** Diabète, médecin généraliste, coordination, prise en charge, formation continue, Diabetes, general practitioner, coordination, management, continuous training

## Abstract

**Introduction:**

Le diabète constitue un important enjeu de santé publique au Maroc et représente un défi auquel les médecins généralistes sont confrontés dans leur pratique quotidienne. Le but de ce travail était de décrire les barrières entravant une bonne prise en charge des patients diabétiques dans les structures de 1ère ligne de la province de khouribga.

**Méthodes:**

Il s'agit d'une étude transversale menée de décembre 2010 à mars 2011, chez les 54 médecins généralistes (MG) exerçants dans les centres de santé de la province. La collecte des données a été réalisée à l'aide d'un questionnaire prétesté et auto administré et la saisie et l'analyse effectuées sur le logiciel SPSS 16.

**Résultats:**

Huit pourcent des MG disposaient de registre informatisé pour le suivi des diabétiques. Les principales barrières à une prise en charge correcte des patients étaient le statut socio-économique faible des patients (94%), leur niveau scolaire bas (86%), le manque de moyens de traitement et de suivi (80%), le nombre insuffisant de diabétologues (80%), le manque de coordination avec les structures de 2ème ligne (74%) et l'insuffisance des séminaires de formation continue (58%).

**Conclusion:**

Les médecins généralistes sont confrontés à de nombreux obstacles concernant la prise en charge des patients diabétiques. L’équipement des centres de santé en moyens diagnostiques et thérapeutiques suffisants, l'amélioration de l'accessibilité des diabétiques aux soins et la formation continue des médecins pourraient être des solutions pour améliorer cette prise en charge surtout devant la pénurie de spécialistes dans notre pays.

## Introduction

Au Maroc, pays en pleine phase de transition démographique, nutritionnelle et épidémiologique [1, 2], la fréquence du diabète est en augmentation avec plus d'un million et demi de diabétiques en 2010. Ce chiffre atteindrait 2,5 millions à l'horizon 2030 [3]. C'est une maladie dont le traitement est à vie, la prévention de ses complications ne va pas seulement améliorer la qualité de vie des patients, mais réduira aussi les coûts de leur prise en charge pour le secteur de santé et les services sociaux (206 millions de Dollars US au Maroc et 367 milliard de Dollars US dans le monde en 2010, soit 12% des dépenses en santé au niveau mondial) [4].

Une prise en charge spécialisée exclusive par les structures de 2ème ligne est impossible vueleur rareté et le nombre réduit de médecins spécialistes dans notre pays. De plus, des études ont montré que la prise en charge du diabète par les structures de 1ère ligne peut égaler celle de 2ème ligne, à condition que les médecins généralistes (MG) portent un intérêt spécial pour le diabète et que les soins soient bien organisés [5, 6].

Les objectifs de notre étude étaient de décrire les barrières entravant une bonne prise en charge des patients diabétiques dans les structures publiques de 1ère ligne et d'identifier les besoins de formation exprimés par les MG afin d'améliorer la qualité des soins délivrés à la province de Khouribga.

## Méthodes

Il s'agit d'une étude d'observation transversale à visée descriptive menée à la province de Khouribga. La population totale de cette province a été estimée en 2009 à 500 000 habitants dont338 000 en milieu urbain et 162 000 en milieu rural [7].

Cette province est dotée de 38 établissements de soins de santé de base publics dont 12 en milieu urbain et 26 en milieu rural, 1Hôpital provincial à la ville de Khouribga et 2 Hôpitaux locaux à Oued-Zem et Bejaad. 54 MG exercent dans le réseau de soins de santé de base de la province et il n'y a qu'un seulmédecin endocrinologue dans le secteur public de la province qui exerce à l'hôpital provincial de Khouribga.

La population étudiée était constitué des 54 médecins généralistes exerçant dans les structures de soins de santé de base de la province de Khouribga dont 4 ont refusé de participer à l’étude se justifiant de ne suivre aucun malade diabétique dans leurs consultations à cause de leur affectation récente. Les données ont été collectées de décembre 2010 à mars 2011 à l'aide d'un questionnaire pré testé auto-administré. Les variables étudiées comprenaient la disponibilité des moyens de prise en charge et de suivi des diabétiques dans le centre de santé, les barrières entravant la prise en charge des patients, le niveau de coordination existant entre les structures de 1^ère^ et 2^ème^ ligne et les besoins en formation continue des MG sur le thème diabète. Les données ont été saisies et analysées sur le logiciel SPSS version 16. Des fréquences absolues et relatives ont été calculées pour les variables qualitatives et les moyennes et écarts types pour les variables quantitatives. Nous avons utilisés le test de Chi deux et le test exact de Fisher pour comparer les pourcentages. Le seuil de signification a été fixé à 5%. Avant leur inclusion dans l’étude, les médecins ont été informés des objectifs de l'enquête et leur consentement oral a été obtenu avant l'administration du questionnaire. Par ailleurs, l'anonymat et le respect de la confidentialité des données ont été assurés.

## Résultats

### Caractéristiques générales de l’échantillon

La moyenne d’âge des médecins interrogés était de 39,5 (±7,3) ans avec un sex-ratio H/F de 1,2. Cinquante deux pour cent parmi eux exerçaient en milieu rural. Le nombre moyen d'années d'exercice était 14,4 (±8,2) ans pour les médecins du milieu urbain et 6,9 (±3,5) ans pour ceux exerçant en milieu rural. Le Tableau 1 résume les caractéristiques des médecins exerçant dans la province de Khouribga selon le milieu d'exercice - 2011 (N = 50)

**Tableau 1:** Caractéristiques des médecins exerçant dans la province de Khouribga selon le milieu d'exercice, Maroc, 2011 (N = 50)

Caractéristiques	Urbain (n = 24)%	Rural (n = 26)%
**Age**		
< 40ans	29,2	92,3
40-49ans	37,5	7,7
≥ 50ans	33,3	0,0
**Genre**		
Masculin	58,3	34,6
Féminin	41,7	65,4

### Disponibilité des moyens de traitement et de suivi des patients diabétiques

**Figure 1:**
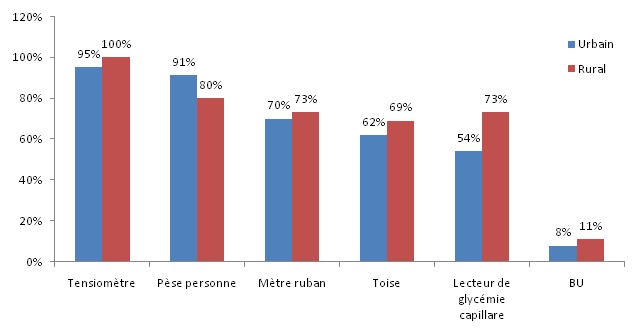
Disponibilité des moyens de suivi des patients diabétiques selon le milieu dans les centres de santé de la province de Khouribga, Maroc, 2011

Parmi les médecins interrogés, 96% ont affirmé avoir un registre pour diabétiques au centre qui était informatisé dans 8,3% des cas. Concernant les moyens de suivi existant au centre de santé, les bandelettes urinaires (BU) étaient le moyen de suivi le plus en manque. La disponibilité des moyens selon le milieu d'exercice est présentée dans la Figure 1. Concernant la disponibilité des médicaments, 60% des médecins interrogés ont jugé que la qualité et la quantité des moyens thérapeutiques existants au centre étaient insuffisantes pour couvrir les besoins de leurs patients diabétiques.

### Les barrières qui entravent la prise en charge correcte des diabétiques

Le statut socio-économique faible des patients, leur niveau scolaire bas, le manque de moyens de suivi et de traitement, le nombre insuffisant de diabétologues, le manque de coordination avec les structures de 2ème ligne et l'insuffisance des séminaires de formation continueont été rapportés comme des problèmes majeurs dans la prise en charge correcte des diabétiques selon les médecins interrogés avec des pourcentages respectifs de 94%, 86%, 80%, 80%, 74% et 58%.

La majorité (96%) des médecins référait leurs patients à l'hôpital provincial lorsqu'ils avaient besoin d'un avis ou d'une prise en charge par l'endocrinologue. Le nombre de patients référés ne dépassait pas 3 par mois dans 78% des cas.Le motif le plus fréquemment évoqué pour cette référence était l'association déséquilibre diabétique et complications (59% des cas). Néanmoins,40% des médecins interrogés ont affirmé n'avoir jamais reçu de feedback concernant les patients référés.

### La formation continue des médecins sur le thème du diabète

Parmi les médecins interrogés, 82% avaient déjà participé au cours de leur carrière à au moins un séminaire de formation continue sur le diabète. Le dernier séminaire remontait à moins de 3mois pour 61% d'entre eux et sa durée était d'une demi-journée dans 51% des cas. Les ¾ de ces formations avaient été organisées par le ministère de la santé et 66% ont porté sur le sujet « Actualités de la prise en charge diabétique ».Les ¾ des médecins ont estimé avoir été satisfaits de ces formations. Le Tableau 2 résume les caractéristiques de la dernière formation à laquelle ont assisté les médecins généralistes de la province de Khouribga ‘ 2011.

**Tableau 2:** Caractéristiques de la dernière formation à laquelle ont assisté les médecins généralistes de la province de Khouribga, Maroc, 2011

Dernière formation	n	%
Date		
< 3mois	25	61
3 mois à 1an	13	32
> 1an	3	7
Durée		
Demie- journée	21	51
Journée	15	37
>1 journée	5	12
Organisateur		
Ministère de la santé	31	75
Association de diabétiques	5	13
Association de médecins généralistes	4	10
Association de médecins endocrinologues	1	2
Sujet		
Actualités de la PEC diabétique	27	66
Traitement médicamenteux diabétiques	9	23
Autres	5	11

Les autres moyens utilisés par les médecins pour leur formation continue étaient la lecture de revues médicales (72%), la consultation de sites internet et des ouvrages médicaux respectivement dans 70% et 66% des cas.La majorité (96%) des médecins ont estimé avoir besoin d'une formation sur la prise en charge du diabète.Les thèmes les plus demandés comme sujet de cette formation étaient la conduite de l'insulinothérapie (87%), le suivi du diabétique (80%), le dépistage des complications (76%) et le traitement par antidiabétiques oraux (70%).

### Influence du milieu d'exercice sur la formation continue des médecins généralistes

Les médecins exerçants en milieu urbain assistaient plus fréquemment (91,7%) aux séminaires de formation continue que ceux du milieu rural (73,1%). En ce qui concerne les autres outils de formation continue, la consultation de sites internet venait en tête (79,2%) des moyens utilisés par les médecins du milieu urbain, alors que ça venait en dernière position chez leurs collègues du milieu rural (61,5%).

Les thèmes souhaités par les médecins comme sujets de futures formations différaient selon le milieu d'exercice.En milieu urbain, les médecins préféraient des sujets portant sur le suivi du diabète (87%) et l’éducation du diabétique (73,9%). En milieu rural, c’était le volet traitement qui venait en tête avec l'insulinothérapie (88,5%) et le traitement par antidiabétiques oraux (76,9%). Le Tableau 3 résume l′influence du milieu d'exercice sur la formation continue des médecins généralistes de la province de Khouribga -2011.

**Tableau 3:** Influence du milieu d'exercice sur la formation continue des médecins généralistes de la province de Khouribga, Maroc, 2011

	Milieu		P
	Urbain n (%)	Rural n (%)	
Participation aux séminaires de formation continue	22 (91,7)	19 (73,1)	0,14
Utilisation de revues médicales	19 (79,2)	17 (65,4)	0,35
Consultation de sites internet	19 (79,9)	16 (61,5)	0,22
Consultation d'ouvrages	15 (62,5)	18 (69,2)	0,76
Besoins de formation	22 (91,7)	25 (96,2)	0,60
**Thèmes souhaités**			
Traitement par ADO*	13 (56,5)	20 (76,9)	0,22
Insulinothérapie	18 (78,3)	23 (88,5)	0,44
Suivi du diabétique	20 (87,0)	18 (69,2)	0,18
Dépistage des complications	18 (78,3)	18 (69,2)	0,53
Education du diabétique	17 (73,9)	12 (46,2)	0,08

* ADO : antidiabétiques oraux

## Discussion

Le diabète constitue un important enjeu de santé publique au Maroc et représente un défi auquel les médecins généralistes sont confrontés dans leur pratique quotidienne [8]. Notre étude est la 1^ère^ à explorer les obstacles à une bonne prise en charge des diabétiques par les médecins généralistes du secteur public de la province de Khouribga. L’échantillon initial était représenté par les 54 médecins généralistes exerçant dans les structures de première ligne de la province, dont 4 ont refusé de participer à l’étude. La population étudiée était jeune, puisque 84% étaient âgés de moins de 50ans avec une moyenne d’âge de 39 (± 7,3) ans et une légère prédominance masculine avec 54% d'hommes. Cinquante deux pourcent des MG exerçaient dans le milieu rural.

Parmi les médecins interrogés, 96% ont affirmé avoir un registre pour diabétiques au centre. Ce registre est un moyen nécessaire pour le suivi de toute maladie chronique [6]. Huit pourcent de ces registres étaient informatisés, ce qui est très en dessous des chiffres relevés dans les études réalisées en Irlande du nord et en Angleterre qui étaient respectivement de 66% et 77% [5, 6]. Un registre informatisé facilite la conservation des données pour un suivi plus facile des patients, et leur exploitation à grande échelle pour des études statistiques ultérieures [9–11]. Les centres de santé ne disposaient pas de tous les moyens de base pour un suivi clinique de l’évolution de la maladie diabétique. Les bandelettes urinaires étaient le moyen de suivi de base le plus en manque au niveau des centres avec seulement 10% des centres qui en disposaient limitant ainsi le diagnostic des cas de protéinurie et de cétonurie [12]. Concernant les moyens thérapeutiques existants au centre de santé, 60% des médecins interrogés estimaient qu'ils étaient insuffisants en qualité et en quantité pour couvrir les besoins de leurs patients diabétiques.

### Les barrières qui entravent la prise en charge correcte des diabétiques

Le statut socio-économique faible et l'analphabétisme des patients, le manque de moyens de suivi et de traitement, le nombre insuffisant de diabétologues, les problèmes de coordination avec les structures de 2^ème^ ligne ainsi que le manque de formation continue des médecins constituaient des problèmes majeurs dans la prise en charge correcte des diabétiques selon les médecins interrogés.

Ces problèmes reflètent les défis que le système de santé marocain se doit de vaincre à savoir les problèmes d'accès aux soins pour les populations rurales et défavorisées. En matière de financement du système de santé, la dépense globale de santé est faible, soit uniquement 5% du PIB (ce qui reste inférieur à celle des pays au développement économique similaire à notre pays, où elle s’élève à 6,4% du PIB en Tunisie et 9,5% en Jordanie). L'insuffisance du financement public et la faiblesse de la couverture par l'assurance maladie engendrent une iniquité du financement de la santé [7, 13]. Par ailleurs, le système de soins souffre de cloisonnements d'ordre fonctionnel et technique entre les niveaux ambulatoire et hospitalier en l'absence d'un cadre légal de régulation et d'un outil de planification temporelle et spatiale de l'offre publique des soins (Carte sanitaire) [13] Enfin, les médecins ont considérés l'analphabétisme comme un des problèmes majeurs pour une bonne prise en charge. En effet, son taux au Maroc a été estimé en 2009 à 39,7% de l'ensemble de la population et à 55,6% en milieu rural. Le niveau élevé de l'analphabétisme résulte, en partie, des défaillances et des déperditions importantes qui affectent le système éducatif [14].

Soixante-trois pour cent des médecins étaient insatisfaits du contact entre eux et l'endocrinologue de l'hôpital provincial, ce qui traduit la pauvreté de la coordination entre les structures de 1^ère^ et 2^ème^ ligne. Un pourcentage similaire a été retrouvé en Irlande du nord (71%) qui donnait comme explication à cette insatisfaction, la relative dispersion des centres et leur situation le plus souvent rurale [5]. Ce qui était aussi le cas dans notre étude.

Plus de la moitié (58%) des centres ne disposaient pas du planning des journées de consultation des médecins spécialistes au niveau de l'hôpital provincial rendant la tache encore plus difficile pour les patients surtout ceux du milieu rural. Une meilleure collaboration entre les généralistes et les diabétologues est nécessaire pour améliorer la prise en charge des diabétiques suivis dans les structures de 1ère ligne [15, 16].

### La formation continue des médecins sur le thème du diabète

Plus des ¾ des médecins avait déjà participé au cours de leur carrière à au moins 1 séminaire ou conférence de formation continue sur le diabète. Cette formation remontait à moins de 3 mois dans 61% des cas. Ce qui souligne l'engagement de la plupart des médecins dans l'amélioration de leur prise en charge diabétique. La durée de la formation était d'une demi-journée dans 51% des cas et elle était organisée par le ministère de la santé dans 73,2% des cas, alors que dans les autres études, c'est l'industrie pharmaceutique qui s'en chargeait [5, 6]. Ceci montre l'intérêt que porte le ministère de la santé à la formation continue des médecins. Néanmoins, la durée de ces formations reste insuffisante car il est difficile de se tenir au courant des développements les plus importants dans le traitement du diabète avec seulement une demi-journée comme l'ont souligné les études similaires irlandaise et anglaise [5, 6]. La consultation de sites internet venait en tête (79,2%) des moyens utilisés par les médecins du milieu urbain, alors que ça venait en dernière position chez leurs collègues du milieu rural (61,5%). Les nouvelles technologies et surtout internet prennent de plus en plus d'importance comme source d'information pour les praticiens et ceci pour plusieurs raisons à savoir l'actualisation permanente, les liens interactifs, les illustrations nombreuses, l'archivage, les bénéfices de l'intelligence collective, l'accessibilité, l'autonomie, la flexibilité et les possibilités illimitées de ce support [17–20]. Les 2/3 des formations auxquelles avaient assistés les médecins portaient sur le sujet « Actualités à propos de la prise en charge diabétique ». Les trois-quart des médecins estimaient être satisfaits de ces formations. Ce qui nous permet de souligner l'importance de ces cours malgré que l'absence de données objectives sur leur valeur dans l'amélioration de la prise en charge diabétique comme l'avait conclue l’étude anglaise [6]. La majorité (96%) des médecins estimaient avoir besoin d'une formation sur la prise en charge du diabète, ce qui montre bien l'intérêt que porte les médecins pour leur formation continue et l'amélioration de leur pratique quotidienne. Les thèmes souhaités par les médecins comme sujet de futures formations différaient selon le milieu d'exercice. En milieu urbain, les médecins préféraient des sujets portant sur le suivi du diabétique (87%) et l’éducation sanitaire (73,9%). En milieu rural, c’était le volet traitement qui venait en tête à savoir la conduite de l'insulinothérapie (88,5%) et le traitement par Antidiabétiques oraux (76,9%). Ceci pourrait s'expliquer par un accès aisé des populations urbaines aux centres de santé rendant un suivi régulier des patients plus facile, alors qu'en milieu rural, le volet traitement prend toute son importance vu l’éloignement des structures sanitaires et le taux d'analphabétisme plus élevé [13].

## Conclusion

Les résultats de notre étude ont permis de mieux connaître les obstacles auxquels les médecins généralistes sont confrontés dans leur prise en charge des patients diabétiques. Les principales barrières étaient le manque de formation continue et l'insuffisance des moyens diagnostiques et thérapeutiques dans les centres de santé. Il est donc indispensable d'organiser des séminaires sur le diabète au profit des médecins généralistes en tenant compte de leurs besoins et de leur milieu d'exercice et de mettre à leur disposition les moyens nécessaires pour leur permettre d'avoir un bon contrôle glycémique chez leurs patients, condition indispensable pour réduire le risque de complications dégénératives du diabète.
